# The Consumption of Nuts is Associated with Better Dietary and Lifestyle Patterns in Polish Adults: Results of WOBASZ and WOBASZ II Surveys

**DOI:** 10.3390/nu11061410

**Published:** 2019-06-22

**Authors:** Anna M. Witkowska, Anna Waśkiewicz, Małgorzata E. Zujko, Danuta Szcześniewska, Witold Śmigielski, Urszula Stepaniak, Andrzej Pająk, Wojciech Drygas

**Affiliations:** 1 Department of Food Biotechnology, Faculty of Health Sciences, Medical University of Bialystok, Szpitalna 37, 15-295 Bialystok, Poland; malgorzata.zujko@umb.edu.pl; 2 Department of Epidemiology, Cardiovascular Disease Prevention and Health Promotion, Institute of Cardiology, Alpejska 42, 04-628 Warsaw, Poland; awaskiewicz@ikard.pl (A.W.); dszczesniewska@ikard.pl (D.S.); wsmigielski@ikard.pl (W.Ś.); wdrygas@ikard.pl (W.D.); 3 Department of Epidemiology and Population Studies, Faculty of Health Sciences, Institute of Public Health, Jagiellonian University Medical College, Grzegórzecka 20, 31-531 Krakow, Poland; urszula.stepaniak@uj.edu.pl (U.S.); andrzej.pajak@uj.edu.pl (A.P.); 4 Department of Social and Preventive Medicine, Faculty of Health Sciences, Medical University of Lodz, Hallera 1, 90-001 Lodz, Poland

**Keywords:** nuts, dietary patterns, lifestyle patterns, adults

## Abstract

In recent years, the concept of the health benefits of synergistic dietary patterns as opposed to individual foods or food constituents has been developed. The aim of this study was to determine whether nut consumption is associated with healthier nutrition and lifestyle. The research was based on complete data obtained during two Polish National Multi-Centre Health Examination Surveys—WOBASZ (2003–2005) and WOBASZ II (2013–2014). Of the 12,946 participants who completed dietary assessments, 299 subjects reported consuming any quantity of whole nuts. A control group of 1184 non-nut consumers from both surveys was randomly selected for the study, with age, gender, study (WOBASZ, WOBASZ II), educational level, and season-related interactions taken into account. In this study, nut consumption was associated with favorable food and lifestyle choices, excluding smoking. Better dietary quality consisted of having a higher Healthy Diet Indicator score, an increased intake of polyphenols and antioxidants, lower intake of red meat, but higher of poultry and fruit, more frequent consumption of antiatherogenic food products, and less frequent consumption of processed meats. There was also greater interest in special diets, such as weight-loss diet. In addition, nut eaters were more physically active in their leisure time. While limited by 24-h recall of nut intake and possible misclassification of nut/non-nut consumer status, this research supports the synergistic health-promoting attitudes of those who were classified as nut consumers.

## 1. Introduction

Nuts are currently recommended as an important component of cardioprotective diets [1,2]. In subjects at high cardiovascular risk, nut consumption is inversely associated with the prevalence of general obesity, central obesity, metabolic syndrome, and diabetes [3,4,5].

The health benefits of nuts are related to their high unsaturated fat content, phytosterols, L-arginine, fiber, polyphenols, calcium, magnesium and potassium, and low sodium content [2,6]. Nuts are characterized by their high nutritional density, but due to their high energy content they should be consumed in limited quantities [7]. In addition to containing nuts, a healthy diet places emphasis on vegetables, fruit, whole grains, legumes, and a reduction in the consumption of animal products, especially red meat [7,8]. Elements of a healthy lifestyle also include physical activity and avoiding smoking [7,8]. A number of studies have pointed to the role that socioeconomic and educational factors play regarding nut consumption [9] and indicate the link between higher nut consumption and lower body mass index (BMI) and lower waist circumference [10,11,12]. Interestingly, nut consumers have better nutrient adequacy and diet quality [13,14,15], which are important in the prevention of cardiovascular disease (CVD). The number of studies which have aimed to determine whether nut intake is related to better nutrition and healthier lifestyle choices is limited [9,15,16,17].

In recent years, the concept of the health benefits of synergistic dietary patterns as opposed to individual foods or food constituents has been developed [18]. This synergy is based on nutritional diversity and the consumption of nutrient-rich foods. The aim of the study was to determine whether nut consumption is associated with healthier habits in areas such as nutrition (i.e., diet quality, atherogenicity of diet, and dietary practices) and lifestyle (i.e., smoking and physical activity).

## 2. Materials and Methods

### 2.1. Study Participants

**Figure 1:**
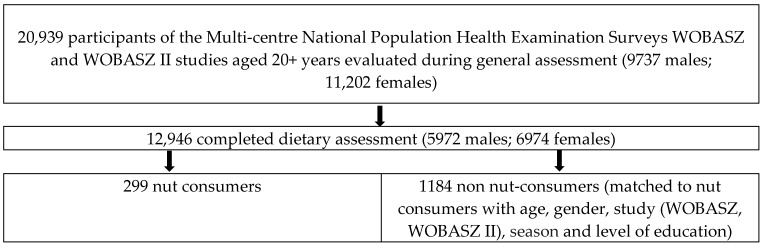
Flowchart of study participants.

The research performed in this work was based on complete data obtained during two Polish National Multi-Centre Health Examination Surveys—WOBASZ (2003–2005) and WOBASZ II (2013–2014)—which are the largest population-based cross-sectional studies conducted in Poland by the National Institute of Cardiology in Warsaw, Poland, in cooperation with five Polish medical universities [19,20,21]. Assumptions and research objectives are described in the WOBASZ manual [22]. The studies collected data on the variables level of education, health status, physical activity at leisure (PAL), and smoking habits, using standard questionnaires developed for the WOBASZ study. A representative sample of 20,939 men and women aged 20–74 years (WOBASZ) and over 20 years of age (WOBASZ II) was evaluated in these studies (Figure 1). The WOBASZ survey protocol was approved by the Bioethics Committee of the National Institute of Cardiology (WOBASZ, no. 708; WOBASZ II, no. 1344).

### 2.2. Control Matching Procedure

It was found during the study that 299 participants from both the WOBASZ and WOBASZ II surveys consumed nuts (Figure 1). In order to determine the relationship between nut consumption, dietary habits, diet quality, and lifestyle patterns, and to exclude age, gender, study (WOBASZ, WOBASZ II) educational level, and season-related interactions, a control group of 1184 non-nut consumers from both surveys was randomly selected for the study group, taking these four factors into account. The MS Excel procedure RANDOM QUOTA SAMPLE was applied while maintaining the structure of gender, age (20–34, 35–49, 50–64, and 65+ years), education (primary, secondary, and university level), and WOBASZ survey (WOBASZ or WOBASZ II) in relation to the sample of nut consumers, taking into account the season of the year.

### 2.3. Data Collection

During the WOBASZ and WOBASZ II surveys, systolic (SBP) and diastolic (DBP) blood pressure measurements were performed. Hypertension was diagnosed if SBP ≥ 140 mmHg and/or DBP ≥ 90 mmHg and/or if antihypertensive drugs were used. Body measurements such as height, body mass, and waist circumference were taken by personnel trained in standard procedures. Body mass index (BMI) was calculated as weight in kilograms divided by the square of height in meters. Central obesity was determined as waist circumference ≥ 102 cm for men and ≥ 88 cm for women. During both surveys, the following laboratory parameters were analyzed: total cholesterol, LDL and HDL cholesterol, triglycerides, and glucose. Biochemical analyses were carried out at the Central Laboratory “Diagnostyka” at the Institute of Cardiology in Warsaw, which has a Center for Disease Control (CDC—Lipid Standardization Program) certificate from Atlanta and a European quality certificate Random International Quality Assessment Scheme (RIQAS).

Harmonized criteria for defining metabolic syndrome (MetS) were adopted [23]. MetS was identified given at least three of the five risk factors: (1) elevated waist circumference of ≥ 94 cm for men and ≥ 80 cm for women, (2) elevated triglycerides (≥ 150 mg/dL–1.7 mmol/L), (3) reduced HDL cholesterol (< 40 mg/dL–1.0 mmol/L for men and < 50–1.3 mmol/L for women), (4) elevated blood pressure (systolic ≥ 130 and/or diastolic ≥ 85 mmHg) or hypertension in an interview, and (5) elevated fasting glucose (≥ 100 mg/dL–5.6 mmol/L) or diabetes in an interview.

Trained nurses collected data on lifestyle factors and diet using standard questionnaires. Smoking status was assessed using three categories—current smokers, past smokers, and never smokers—with smoking defined as having a habit of smoking at least one cigarette a day. Physical activity at leisure was classified using three levels—low, medium, and high. A low-level PAL was defined as when there was no physical activity—for example, jogging, cycling, swimming, and gardening—for at least 30 min a day, or there was only occasional activity (occurring once a week, several times a month, or several times a year). A middle-level PAL was defined as when, for example, jogging, cycling, swimming, and gardening lasted for at least 30 min a day every second or third day. A high-level PAL was defined as physical activity as described above occurring every day or almost every day.

### 2.4. Nutritional Assessment

#### 2.4.1. 24-h Dietary Recall Method

Of the 20,939 participants, 12,946 completed a nutritional assessment collected using a single 24-h recall method (Figure 1). The size of food portions was determined on the basis of photo albums of food provided by the National Food and Nutrition Institute in Warsaw [24]. During the dietary recall, participants were asked whether their diet on a given day was typical of their usual nutrition. Individuals who described their diet as not typical were excluded from further research.

#### 2.4.2. Determination of Nut Consumption

Total intake of nuts was determined on the basis of a 24-h dietary recall and was expressed in g/day. Of the 12,946 participants who completed the dietary assessment (Figure 1), 299 subjects reported consuming any quantity of whole nuts, namely, almonds, hazelnuts, peanuts, pistachios, or walnuts, as individual nuts or in combination. Information on nut preparation (salted, unsalted, roasted, or unroasted) was not collected during the interview. This group was defined as ‘nut consumers’. The consumption of nuts in food products (e.g., in candy bars or cookies) or other hidden sources was not taken into account. The consumption of nut butter was not included in the databases of both WOBASZ surveys. Coconuts were not taken into account due to their different nutritional composition. Participants who reported consuming a quantity of zero nuts in their 24-h dietary recall were classified as ‘non-nut consumers’.

#### 2.4.3. Diet Quality Assessment

Quality of diet was determined on the basis of an assessment of Healthy Diet Indicator (HDI) scores, which were employed with reference to the World Health Organization (WHO) dietary guidelines [25] and were described by Fransen et al. [26]. In brief, HDI is based on the intake of six nutrients—saturated fatty acids (% total energy (TE)), polyunsaturated fatty acids (%TE), dietary cholesterol (mg/day), protein (%TE), fiber (g/day), and free sugars (%TE))—and the fruits and vegetables food group (g/day), within the recommended range [25]. Compliance with WHO recommendations was awarded one point and noncompliance zero points. The final HDI score was the sum of all components, from zero (minimal adherence) to seven (maximal adherence). Nutrient intakes in this study were determined on the basis of 24-h recalls. On the basis of a range of types of food consumption, the nutritional value of each patient’s diet was calculated using Polish national food composition tables [27].

#### 2.4.4. Estimation of Dietary Total Antioxidant Capacity and Dietary Polyphenol Intake

Dietary total antioxidant capacity (DTAC) and dietary polyphenol intake (DPI) were calculated as described previously [28,29]. Briefly, the DTAC was evaluated using databases of the ferric-reducing antioxidant potential (FRAP) of foods [30,31,32] and DPI was calculated using databases of the total polyphenol contents of foods [30,31,33]. DTAC and DPI were determined by multiplying the daily intake of particular foodstuffs by the antioxidant activity or polyphenol contents of these foodstuffs, respectively.

#### 2.4.5. Food Frequency Questionnaire (FFQ)

The usual eating frequency of various food products during the last two to three months was measured. The questionnaire contained 18 groups of food products: (1) milk, kefir, yoghurt, and milk drinks; (2) cottage cheese and curd cheese; (3) hard cheese and processed cheese; (4) beef, pork, veal, lamb, and offal (variety meat); (5) poultry and poultry products; (6) beef, pork, and veal products; (7) fresh, canned and smoked fish; (8) eggs; (9) butter; (10) soft margarines; (11) mixed fats; (12) pork fat and lard; (13) oils; (14) boiled vegetables; (15) raw vegetables; (16) dry leguminous plants; (17) fruit; and (18) fruit and vegetable juices. Food consumption frequency categories (number of days per month) were calculated using daily consumption (30 days per month), four to six times a week (an average of 21.4 days per month), two to three times a week (an average of 10.7 days per month), once a week (an average of 4.3 days per month), less frequently than once a week (one to two times a month, an average of 1.5 times a month), and zero consumption (zero times a month). The frequency of consumption questionnaire did not consider nuts.

#### 2.4.6. Determination of Atherogenic and Antiatherogenic Food Consumption

The number of days on which atherogenic and antiatherogenic products were consumed was determined on the basis of a food frequency questionnaire. The following were considered to be atherogenic products: red meat (processed and unprocessed), hard cheese, butter, and lard. Antiatherogenic products considered were fish (fresh, preserved, and smoked), vegetables (raw and cooked), fruit, and legumes. Since bread was included in the questionnaire as a whole, with no differentiation between white and dark breads, it was not presented in the results.

The atherogenic/antiatherogenic ratio was calculated by dividing the frequency of atherogenic product consumption (in days) by the frequency of antiatherogenic product consumption (in days).

#### 2.4.7. Determination of Dietary Practices and Self-Assessment of Nutrition

Nutrition practices and self-assessment of subjects were determined on the basis of a standard questionnaire developed for the WOBASZ Survey. The participants answered ‘yes’ or ‘no’ to questions concerning the regular addition of salt to meals, the removal of visible fat from meat and cold cuts, the removal of poultry skin, dieting (low-fat diets, diabetic diets, weight-loss diets, and other diets), and self-assessment in terms of whether their nutrition was appropriate. The answers in each category were summed up and the results were reproduced as a percentage of the participants who answered ‘yes’ to the questions.

### 2.5. Statistical Analysis

Statistical analyses were carried out using SAS software version 9.2 (SAS Institute Inc., Cary, NC, USA). Parameters of descriptive statistics were used to describe continuous variables, and for categorized variables, percentages of individual values were used. Due to the lack of a normal distribution (which was tested using the Shapiro–Wilk test), the rank-sum Wilcoxon test was used to compare continuous variables and the chi-square test was used for categorical variables across the categories of nut consumers and non-nut consumers. Due to the small number of participants (18 individuals) with a stroke in the nut-eating group, the exact Fisher test was used to calculate significant differences between the nut consumer and non-nut consumer groups. A *p*-value less than 0.05 was considered statistically significant.

## 3. Results

In order to exclude the impact of variables that may have affected levels of nut consumption, participants were matched according to season, age, gender, and educational level. Hence, no statistical differences were observed between nut consumers and non-nut consumers in relation to these characteristics (Table 1). Additionally, following the application of the matching procedure, it was found that there were no statistical differences observed for smoking habits and health status, i.e., the prevalence of hypertension, myocardial infarction, stroke, diabetes, hypercholesterolemia, central obesity, and metabolic syndrome. In nut consumers, the level of physical activity at leisure was higher (*p* = 0.026). Dietary practices differed between nut consumers and non-nut consumers in terms of dieting. A significantly larger group of nut consumers was on a low-fat, low-cholesterol and diabetic diet, or a weight-loss diet or other diet (*p* = 0.0394). Slightly higher percentages of nut consumers removed visible fat from meat and cold cuts or/and removed skin from poultry (52.51%) and considered their nutrition as appropriate (58.19%) compared to non-nut consumers (48.48% and 53.63%, respectively). In turn, a higher percentage of non-nut consumers (21.88%) regularly added salt to meals compared to nut consumers (18.39%). The above differences were not statistically significant.

In this study, nuts were consumed by 299 (2.31%) participants. The quantities consumed ranged from 30 to 70 g, with an average of 56 g (Table 2). Nut consumers had a significantly higher intake of energy, poultry, fruit, polyphenols, and antioxidants than non-nut consumers (*p* < 0.001). They also ate less red meat (*p* < 0.0198). In addition, nut consumers scored significantly better on a seven-point HDI scale, with a median of 4 points compared to a median of 3 points for non-nut consumers.

The results in Table 3 show that nut consumers consumed fresh fruit more often and had a significantly more infrequent intake of processed red meat than non-nut consumers (*p* = 0.0009). In addition, the overall frequency of consumption of antiatherogenic products was higher in nut consumers (66.2 versus 62.2; *p* = 0.0084) and they had significantly lower atherogenic/antiatherogenic food ratios than non-nut consumers (*p* = 0.0009).

## 4. Discussion

Unlike in other studies, in which nut consumers were compared with the entire studied population of non-nut consumers, this study used a gender, age, and education matching procedure. This approach resulted in a better match between groups of non-nut eaters and nut eaters in order to better reflect the attitude of nut consumers and non-nut consumers with regard to several aspects of nutrition and lifestyle. This entailed an adjustment in terms of factors associated with body mass and health status, which can be confounding factors. For example, results may be affected by health and age, e.g., there may be more unhealthy or elderly people in the group of non-nut consumers, who usually eat less food, including nuts. In this study, the same prevalence of diseases was observed in both groups, including hypertension, myocardial infarction, stroke, diabetes, hypercholesterolemia, central obesity, and metabolic syndrome, which may have resulted from the selection of a group according to several common factors such as age, gender, and educational level. Furthermore, the study design does not take into account the regularity of nut consumption, but merely reflects the prior 24-h recall of nut consumption, which may not accurately reflect usual nut intake. Usually health effects can be observed with regular consumption of a particular food or food ingredient. However, a positive attitude towards one type of healthy food may entail a better attitude towards more healthy foods or behaviors, which may result in better health. Therefore, this study sought aspects of nut consumption in relation to other dietary or lifestyle habits.

An important step in this study was to define a group of ‘nut consumers’. Generally, epidemiological studies use total nut consumption, including nut butters and hidden nut sources, in food products and dishes. In comparison to total nut consumers, whole-nut consumers have been shown to make more informed choices regarding nut consumption [17]. In this regard, a similar approach was applied as in the US study, which identified a group of out-of-hand nut (OOHN) consumers [17].

Taking into account aggregate data from the WOBASZ and WOBASZ surveys for one 24-h dietary recall, only 2.31% of participants, with regard to the entire population, consumed nuts. This is less than other studies have indicated [9,10,34], but similar to Sweden, where 2.5% subjects consumed nuts according to a 24-h recall [34]. However, in the case of the WOBASZ survey, which was carried out between 2003 and 2005, this percentage was 1.82%; ten years later with the WOBASZ II survey it was 2.98%, which can be explained by economic and social changes. During these 10 years, knowledge about nuts and recommendations in this area have changed, which could have had an impact on the higher frequency of nut consumption in the second study [35]. However, compared to other European countries, this is still less. A higher percentage of daily nut consumption was reported in the European Prospective Investigation into Cancer and Nutrition (EPIC) for nine European countries (Denmark, France, Germany, Greece, Italy, Netherlands, Norway, Spain, and the United Kingdom) [34] and in other research conducted in Croatia, Argentina, and New Zealand [9,10,11,36]. Nutrition patterns in Central Europe may differ in this respect [11]. The EPIC study indicated an increasing gradient in nut consumption from northern to southern countries [34]. Nut consumption may also depend on dietary patterns. For example, higher consumption of nuts was recorded for vegetarians, vegans, and other health conscious populations as compared to omnivores [11,34]. The determination of the size of a nut consumer group depends on how the data are collected and how ‘nut consumers’ are defined. In a Mediterranean population (PREDIMED-PLUS study) using a food frequency questionnaire, 82% of nut consumers were identified by any amount of nuts consumed against non-nut consumers who reported consuming zero quantity of nuts [37]. For comparison purposes, it is also important to consider whether nut consumption was measured as a total, including hidden nut sources, or only using whole nuts. In the present study, only the consumption of whole nuts was taken into account, which is one of the reasons why our results may be lower than those in other studies. The average nut portion size in our study was 56 g/day, with nut portion sizes ranging from 30 g/day to 70 g/day. In the EPIC study, which also used 24-h recalls to measure nut consumption in 10 European countries, the average portion size of nuts was 30.8 g/day [10]. The highest intake was observed in Norway (43.2 g/day) and the lowest in Sweden (22.7) g/day. In terms of portion sizes, our results are very satisfactory and they are even higher than the recommended intake of 30–42 g/day [38,39]. Although nuts are nutritionally dense, they also have a high energy content, which, due to the large portion volumes recorded in this study, can be even higher in energy. Many studies, however, stress that eating nuts does not increase the risk of obesity [10,11,12]. However, with regard to the beneficial nutritional properties of nuts, it should still be kept in mind that in our study only a small percentage of participants consumed nuts—a smaller percentage than that in other countries.

In this study, nut consumers were characterized by having better dietary habits than their counterparts who did not consume nuts. This finding is consistent with the results of other studies [9,10,17,37]. Overall, dietary quality in this study was measured in various ways. Firstly, HDI scores were used in this study. HDI is one of several nutrition evaluation indicators that have been used in population studies [40,41]. HDI reflects the dietary recommendations of WHO for the prevention of chronic diseases [25]. In the present study, HDI scores of nut consumers were significantly higher by one point in the 7-point scale compared to those of non-nut eaters (with a median of four versus three). For comparison, in PREDIMED-PLUS study, nut consumers achieved higher dietary quality scores such as Mediterranean diet score (MDS), carbohydrate quality index (CQI), and fat quality index (FQI) [37]. PREDIMED-PLUS study and our study showed that nut consumption can be a marker of higher diet quality.

Another method used to assess diet quality in this study was to determine the intake of foods that are recommended or restricted in a healthy diet. Nutritional recommendations include a minimum intake of 400 g of fruit and vegetables. In the case of our own research, these recommendations were met for both groups. However, nut consumers consumed both vegetables and fruit more, in total consuming 548 g, with non-nut consumers consuming 442 g. Although the quantities of fruit and vegetables in this study were higher for nut consumers, significant differences were found only for fruit intake in relation to non-nut consumers. Moreover, for nut consumers, a significantly higher consumption of poultry and a significantly lower consumption of red meat in comparison to non-nut consumers were observed. It is widely known from various studies that the excessive consumption of red meat is a factor in the development of several chronic diseases, such as CVD and cancers [42,43,44,45], with the same not observed for white meat [44]. These findings have been reflected in dietary guidelines with an aim to reduce red meat consumption in favor of other protein sources, especially plant food and lean meats [46,47]. Such recommendations could probably have contributed to lower red meat consumption among nut consumers in our study. As in the present study, a higher intake of fruit and vegetables and a lower consumption of meat have been observed in African-American nut-consuming women [12]. In our study, a higher consumption of fruit and vegetables may translate into a significantly higher intake of dietary polyphenols and antioxidants. Various mechanisms of polyphenol protection against CVD are known [48], and therefore frequent consumption of fruit and vegetables, which are among the best dietary polyphenol sources [49], should be promoted.

In the current study, the consumption frequency of various food products was also examined. However, in both WOBASZ surveys, the consumption frequency was not measured for nuts. Taking into account the available data, it was concluded that nut consumers ate processed red meat less frequently and ate fresh fruit more often compared to non-nut consumers. In addition to red meat, an increased consumption of processed meat is another risk factor for CVD [44]. It was also found that nut eaters consumed significantly more antiatherogenic products in comparison to non-nut eaters. This was reflected in the ratio of atherogenic to antiatherogenic products, which was significantly lower among nut eaters. However, these results are debatable in light of other studies which have concluded that a high consumption of fruit and vegetables cannot counterbalance the negative impact of red meat consumption with respect to CVD [41].

Nuts are recommended in health-promoting diets such as the Mediterranean, Dietary Approaches to Stop Hypertension (DASH), and portfolio diets. In the present study, respondents were asked about their use of special diets such as low-fat diets, low-cholesterol or diabetic diets, weight-loss diets, or other diets (%). The percentage of subjects who followed a diet was higher for the group of nut consumers than for the group of non-nut eaters. However, more detailed data on using diets (i.e., reasons and for how long) was not collected in our study. This study showed for the first time a link between the use of special diets and the consumption of nuts.

In the current study, energy intake was higher among nut consumers compared to those who did not consume nuts, as seen in studies carried out in the USA and New Zealand [10,17]. Interestingly, a higher energy intake in nut consumers was observed in different age groups (children and adults) [17]. Our results show that energy intake was very close to that given in New Zealand data for the adult population. The energy consumption in our study averaged 2281 kcal/day for nut consumers and 2044 kcal/day for non-nut consumers. By comparison, in New Zealand research it was 2266 kcal and 1949 kcal, respectively.

In this study, as in other studies on nut consumption, physical activity was measured [9]. Relja et al. [9] found less intensive daily physical activity levels in frequent nut consumers, which were explained by the intensive physical activity at work of less educated physical workers, who consume nuts less frequently. In our study, only physical activity at leisure was measured, which better reflects the health-promoting attitudes of the subjects. We found that a higher percentage of nut consumers had medium and high PAL scores and in the group of non-nut consumers the highest percentage demonstrated low PAL.

A reason for concern is the similar level of smoking seen in both groups. Earlier studies had indicated a lower percentage of current smokers in groups that often consumed nuts [9,10]. The current study did not show significant differences regarding smoking, but a slightly higher percentage of nonsmokers and ex-smokers were found for nut consumers. As in other studies, in our study the percentage of current smokers was lower for nut consumers than for non-nut consumers (22% versus 26%). However, this difference was not statistically significant.

The main limitation of this study was its cross-sectional design, because of which causal inferences cannot be made. It is not possible for us to determine whether eating nuts is a result of increased nutritional awareness and informed dietary choices.

Another limitation is that a single 24-h recall method was applied, which may not reflect a typical diet. In order to reduce the possibility of bias, participants were asked if their diet in a 24-h recall was typical of their usual nutrition. Those who described their diet as atypical were excluded from the study. However, it is still not entirely possible to exclude nut/non-nut consumer misclassification bias. It is possible that some nut consumers did not consume nuts that day. On the other hand, the occasional consumption of nuts on a given day may have led to misclassification of individuals who normally do not eat nuts as nut consumers.

The limitation was also that there was no distinction between how nuts were prepared for consumption, whether they were salted or not. In connection with the health implications of salt consumption, especially the possibility of developing cardiovascular diseases, it should be reduced in the diet. However, in the study, despite the finding of more favorable dietary habits among nut consumers, it was not found that nut consumers and non-nut consumers differed in terms of sodium intake and percentage of participants adding extra salt to dishes. In the case of salted nuts, however, studies have shown that they retain their cardioprotective properties even during dry roasting and light salting and are therefore recommended as part of a heart-healthy diet [50]. For the sake of accuracy it should be added that the majority of nut eaters in our study, 63%, consumed walnuts and hazelnuts, which in Poland are traditionally sold unroasted and unsalted.

An advantage of this study was its population-representative character, which allowed for comparison of nut consumption in Poland to that in other countries. Also, using further analyses, this study eliminated the influence of factors such as gender, age, level of education, and season through appropriate selection, resulting in more homogenous study groups. The population of individuals classified as nut consumers was small compared to the total population participating in the study. Therefore, in order to reduce the influence of various factors on the results of the study, a proper selection of non-nut eaters by a matching procedure was made based on factors that may have had the greatest influence.

Various studies have not taken into account quality of the diet but rather nutrient intake, meaning that they have difficulties in explaining the reasons behind the relationship between eating nuts and nutrient content. A better-quality diet can facilitate the beneficial health effects observed in nut consumers. The advantage of the current study is that it explains these relationships by means of categories of food products that are eaten more frequently and in larger quantities for a group of nut consumers.

## 5. Conclusions

In this study, nut consumption was associated with favorable food and lifestyle choices, excluding smoking. Better dietary quality consisted of having a higher Healthy Diet Indicator score, an increased intake of polyphenols and antioxidants, lower intake of red meat, but higher of poultry and fruit, more frequent consumption of antiatherogenic food products, and less frequent consumption of processed meats. There was also greater interest in special diets, such as weight-loss diet. In addition, nut eaters were more physically active in their leisure time. While limited by 24-h recall of nut intake and possible misclassification of nut/non-nut consumer status, this research supports the synergistic health-promoting attitudes of those who were classified as nut consumers.

## Figures and Tables

**Table 1:** Descriptive statistics of the studied group.

	Nut Consumers *N* = 299		Non-Nut Consumers *N* = 1184		
Characteristics	mean ± SD	median (25–75 percentile)	mean ± SD	median (25–75 percentile)	*p*
Age (years)	44.1 ± 14.1	43.0 (33–55)	43.2 ± 14.0	43.0 (31–54)	0.3421
	%		%		
Gender (%)					0.974
men	45.27		45.48		
women	54.73		54.52		
Age (%)					0.732
20–44 years	51.52		53.98		
45–64 years	40.74		39.00		
65+ years	7.74		7.02		
Level of education (%)					0.904
under middle	27.76		28.80		
middle	42.47		41.13		
university	29.77		30.07		
Smoking status (%)					0.336
current smokers	22.07		26.02		
past smokers	24.75		22.35		
never smokers	53.18		51.63		
BMI [kg/m^2^] (%):					0.755
underweight (BMI<18.5)	1.37		1.39		
normal (BMI 18.5–24.99)	42.47		42.53		
overweight (BMI 25–29.99)	37.67		35.04		
obese (BMI>30)	18.49		21.04		
PAL (%)					0.026
low level	34.78		43.34		
middle level	32.44		27.40		
high level	32.78		29.26		
Diseases (%)					
Hypertension	33.22		33.48		0.934
Myocardial infarction	3.01		2.70		0.774
Stroke	1.68		1.11		0.386*
Diabetes	7.67		6.71		0.658
Hypercholesterolemia (%)	61.86		62.71		0.789
Central obesity (%)	29.69		32.04		0.439
Metabolic syndrome (%)	27.76		28.38		0.832
Regular adding extra salt during the meal (%)	18.39		21.88		0.1881
Removal of visible fat from meat and cold cuts, removal of poultry skin (%)	52.51		48.48		0.2131
Special diets:					0.0394
low-fat, low-cholesterol, or diabetic diet (%)	7.36		5.15		
weight-loss diet (%)	1.67		0.84		
other diet (%)	3.68		1.77		
Recognizing their nutrition as appropriate (%)	58.19		53.63		0.2976

*—Due to the small number of participants (18 individuals), the results of the Fisher’s exact test are given. SD—standard deviation.

**Table 2:** Energy, food intake, and diet quality in nut-consumers vs. non-nut consumers.

	Nut Consumers *N* = 299		Non-Nut Consumers *N* = 1184		
Intake	mean ± SD	Median (25–75 percentile)	mean ± SD	Median (25–75 percentile)	*p*
Energy (kcal/d)	2281 ± 888	2110 (1659–2755)	2044 ± 997	1872 (1391–2456)	<0.0001
Nut consumption (g/d)	56.00 ± 52.8	30.0 (30.0–70.0)	0 ± 0	0 (0–0)	<0.0001
Red meat (processed, unprocessed) (g/d)	101.5 ± 115.8	60 (0–160)	123.4 ± 157.6	85 (20–184)	0.0198
Fish (g/d)	24.5 ± 66.6	0 (0–0)	19.2 ± 64.6	0 (0–0)	0.0593
Poultry (processed, unprocessed) (g/d)	67.8 ± 108.6	0 (0–120)	53.1 ± 96.8	0 (0–75)	0.0286
Wholemeal bread (g/d)	30.3 ± 58.0	0 (0–50)	27.6 ± 57.0	0 (0–30)	0.2743
Vegetables (g/d)	257.2 ± 189	230 (128.1–350)	235.2 ± 172.7	204.2 (113.8–320.1)	0.0750
Fruits (g/d)	290.5 ± 254.7	250 (110–400)	206.5 ± 241	150 (0–310)	<0.0001
Legumes (g/d)	3.1 ± 12.7	0 (0–0)	4.1 ± 19.2	0 (0–0)	0.9006
Tea (g/d)	341.6 ± 259.5	250 (200–500)	328.8 ± 263.4	250 (200–500)	0.3075
Coffee (g/d)	180.1 ± 179.8	200 (0–250)	182.5 ± 198.8	200 (0–250)	0.9450
Alcohol(pure ethanol g/d)	2.7 ± 9.7	0 (0–0)	3.7 ± 17.7	0 (0–0)	0.9329
Sodium (mg/d) **	1903 ± 1051	1645 (1177–2423)	2026 ± 1273	1706 (1168–2545)	0.3254
DPI (mg/d)	2907.2 ± 1212.7	2677.9 (2118.7–3515)	2040.9 ± 911.4	1951.9 (1402.4–2487.7)	<0.0001
DTAC (mmol/d)	29.3 ± 26.4	21.3 (13.4–35.8)	12 ± 5.9	11.4 (8.0–15.3)	<0.0001
HDI (points)	3.9 ± 1.3	4 (3–5)	3.2 ± 1.3	3 (2–4)	<0.0001

** Excludes sodium consumed as extra salt or in homemade dishes. In the case of processed food, the amount of salt specified in the recipe has been added.

**Table 3:** Frequency of consumption (according to number of days a month) of atherogenic and antiatherogenic food products.

	Nut Consumers *N* = 299		Nut non-Consumers *N* = 1184		
Monthly frequency of intake	mean ± SD	Median (25–75 percentile)	mean ± SD	Median (25–75 percentile)	*p*
Atherogenic foods (sum)	53.1 ± 27.6	54.4 (30.0–72.2)	56.8 ± 25.1	55.7 (39.4–73.3)	0.0501
Antiatherogenic foods (sum)	66.2 ± 23.6	66.4 (50.8–85.0)	62.2 ± 23.5	62.1 (46.5–77.9)	0.0084
Atherogenic/antiatherogenic food ratio	1.01 ± 1.42	0.8 (0.5–1.3)	1.19 ± 1.25	0.9 (0.6–1.3)	0.0009
Hard cheese (days/month)	10.6 ± 9.1	10.7 (4.3–10.7)	10.6 ± 9.07	10.7 (4.3–10.7)	0.9985
Red meats unprocessed (beef, pork, veal, lamb, organ meats) (days/month)	12.0 ± 9.0	10.7 (4.3–21.4)	12.73 ± 8.67	10.7 (4.3–21.4)	0.1055
Processed meats (pork, beef, veal) (days/month)	13.1 ± 9.5	10.7 (4.3–21.4)	15.2 ± 9.7	10.7 (10.7–21.4)	0.0009
Butter (days/month)	15.0 ± 13.7	10.7 (0.0–30.0)	15.8 ± 13.5	21.4 (0.0–30.0)	0.4308
Lard (days/month)	2.38 ± 5.46	0 (0.0–1.5)	2.59 ± 5.43	0 (0–1.5)	0.1815
Fish, fresh, preserved, smoked (days/month)	5.75 ± 5.02	4.3 (1.5–10.7)	5.44 ± 4.73	4.3 (1.5–4.3)	0.4689
Vegetables, cooked (days/month)	15.2 ± 9.9	10.7(10.7–21.4)	14.82 ± 9.91	10.7 (10.7–21.4)	0.5334
Vegetables, raw (days/month)	17.36 ± 9.97	21.4 (10.7–30.0)	16.22 ± 10.55	10.7 (10.7–30.0)	0.0875
Legumes (days/month)	3.12 ± 3.76	1.5 (1.5–4.3)	3.16 ± 4.5	1.5 (1.5–4.3)	0.2774
Fruit, fresh (days/month)	24.75 ± 8.2	30 (21.4–30.0)	22.59 ± 9.67	30 (10.7–30.0)	0.0009
